# The prevalence, grouping, and distribution of stressors and their association with anxiety among hospitalized patients

**DOI:** 10.1371/journal.pone.0260921

**Published:** 2021-12-06

**Authors:** Patricia K. Palmer, Kathryn Wehrmeyer, Marianne P. Florian, Charles Raison, Ellen Idler, Jennifer S. Mascaro

**Affiliations:** 1 Department of Spiritual Health, Emory University Woodruff Health Sciences Center, Atlanta, Georgia, United States of America; 2 Department of Family and Preventive Medicine, Emory University School of Medicine, Atlanta, Georgia, United States of America; 3 Graduate Division of Religion, Emory University, Atlanta, Georgia, United States of America; 4 School of Human Ecology, University of Wisconsin-Madison, Madison, Wisconsin, United States of America; 5 Department of Sociology and Rollins School of Public Health, Emory University; Atlanta, Georgia, United States of America; Medical University of Vienna, AUSTRIA

## Abstract

Anxiety is prevalent among hospital inpatients and it has harmful effects on patient well-being and clinical outcomes. We aimed to characterize the sources of hospital distress and their relationship to anxiety. We conducted a cross-sectional study of inpatients (n = 271) throughout two Southeastern U.S. metropolitan hospitals. Participants completed a survey to identify which of 38 stressors they were experiencing. They also completed the State Trait Anxiety Inventory six-item scale. We evaluated the prevalence of stressors, their distribution, and crude association with anxiety. We then used multivariate logistic regression to estimate the association between stressors and clinically relevant anxiety, with and without adjusting for demographic variables. We used factor analysis to describe the interrelationships among stressors and to examine whether groups of stressors tend to be endorsed together. The following stressors were highly endorsed across all unit types: pain, being unable to sleep, feelings of frustration, being overwhelmed, and fear of the unknown. Stressors relating to isolation/meaninglessness and fear/frustration tend to be endorsed together. Stressors were more frequently endorsed by younger, female, and uninsured or Medicaid-insured patients and being female and uninsured was associated with anxiety in bivariate analysis. After controlling for the sources of distress in multivariate linear analysis, gender and insurance status no longer predicted anxiety. Feelings of isolation, lack of meaning, frustration, fear, or a loss of control were predictive. Study results suggest that multiple stressors are prevalent among hospital inpatients and relatively consistent across hospital unit and disease type. Interventions for anxiety or emotional/spiritual burden may be best targeted to stressors that are frequently endorsed or associated with anxiety, especially among young and female patients.

## Introduction

Hospital inpatients experience high levels of stress and anxiety, which can increase symptom severity and disability and lead to longer, more costly hospital stays and more likely readmission [1–4]. However, there are remarkably few interventions designed to treat anxiety among hospitalized patients, in part because of a paucity of research to understand the emotional and contextual factors associated with anxiety in this vulnerable population [5]. Given the importance of anxiety on health outcomes for hospitalized patients, understanding the stressors and local factors that are associated with anxiety is crucial for designing and tailoring interventions to reduce anxiety and for identifying ways to promote patient well-being.

Risk factors for anxiety are relatively well-characterized among select inpatient populations such as surgical or cardiac patients [6–9]. Previous studies have shown sociodemographic characteristics such as age and gender to be associated with anxiety in multiple patient populations, including those hospitalized with chronic illness [10] or cancer [11], under palliative care [12], or receiving treatment in the emergency department [13]. Additionally, anxiety has been found to be associated with specific stressors in hospitalized patients, for example insufficient sleep, pain, inadequate explanation of the treatment, separation from family, uncertainty, loss of control, fear, and impaired body image among pre- and post-surgical patients [5, 7–9, 14] and physical symptoms, financial instability, social support or isolation, shame, and stress among cancer patients [15, 16]. However, hospital-based approaches have been impeded by the fact that few studies have examined variables associated with anxiety within and across a more inclusive and heterogenous inpatient population. This knowledge gap is problematic for at least three reasons. First, it makes it more challenging to develop interventions or policies targeted to the broader inpatient population (for example [17]). While developing and adapting interventions for the needs of specific patient populations is crucial, there are some scenarios in which the ability to create an intervention or policy change for the hospital experience writ large is necessary. For example, hospital-wide interventions have been shown effective in screening for and addressing domestic violence and nutritional needs, issues that are present in patient populations across the hospital [18, 19]. Further, interventions such as music therapy [20, 21] and mindfulness [17] have been effectively deployed hospital-wide to reduce anxiety and distress across multiple units and disease types. Hospital-wide stressors could be similarly addressed. Second, and related to the first, there are hospital workforce staff who work across units (for example, hospital chaplains) and for whom a more general and less siloed understanding of hospital distress is important because care is provided to patients across the hospital who experience similar types of distress and global interventions are often brought to bear [22, 23]. There is evidence that clinician-led interventions, compared with self-administered approaches, are more effective in reducing anxiety [5]. Understanding the prevalence and distribution of stressors associated with anxiety will be crucial toward deploying clinician intervention. Third, although anxiety is common among inpatients, much of this distress is found in subclinical levels of anxiety that may not meet criteria for psychiatric intervention. To improve the hospital experience for all patients, it is necessary to gain an understanding of the factors that increase anxiety as well as the prevalence and distribution of these sources of distress.

As part of a larger initiative to develop and evaluate interventions for inpatient distress, this cross-sectional study had three objectives. The first was to describe the prevalence of stressors among general hospital inpatients and their distribution across demographics and unit type. The second was to use factor analysis to examine whether stressors associate in patterns of superordinate themes or groups. The third was to examine how stressors were related to anxiety across the hospital inpatient population.

## Methods

### Study design and recruitment

Participants for this cross-sectional study were recruited between August 2017 and January 2018 from two acute-care hospitals in a major metropolitan area in the southeastern United States: Emory University Hospital and Emory University Hospital-Midtown in Atlanta, Georgia. Patients were eligible for inclusion in the study if they were at least 18 years of age, English-speaking, and receiving care on an inpatient unit. Patients were excluded if they were cognitively impaired, on a ventilator, or were in a room requiring enteric precautions or airborne precautions (use of an N-95 mask requiring fit-testing) to enter.

Units were selected for each day’s recruitment efforts to achieve widespread and proportional coverage throughout all inpatient unit types. Each day, patient recruitment within a single unit was determined by generating a room randomization schedule using an on-line random number generator. During recruitment, study team personnel visited hospital rooms in the randomly generated order. If a patient was asleep, out of the room, or engaged in discussion with medical staff during the initial recruitment attempt, researchers made a maximum of two additional recruitment attempts within the same day.

If a patient was able and willing to participate, survey data were collected via tablet at the patient’s bedside (survey questions can be found in the S1 File). The researcher read all survey questions and recorded patients’ responses, which were verbally confirmed with the patient. Data were collected and managed using REDCap (Research Electronic Data Capture) electronic data capture tools hosted at Emory University. The study was approved by the Emory University institutional review board, and all participants provided written, informed consent.

**Fig 1:**
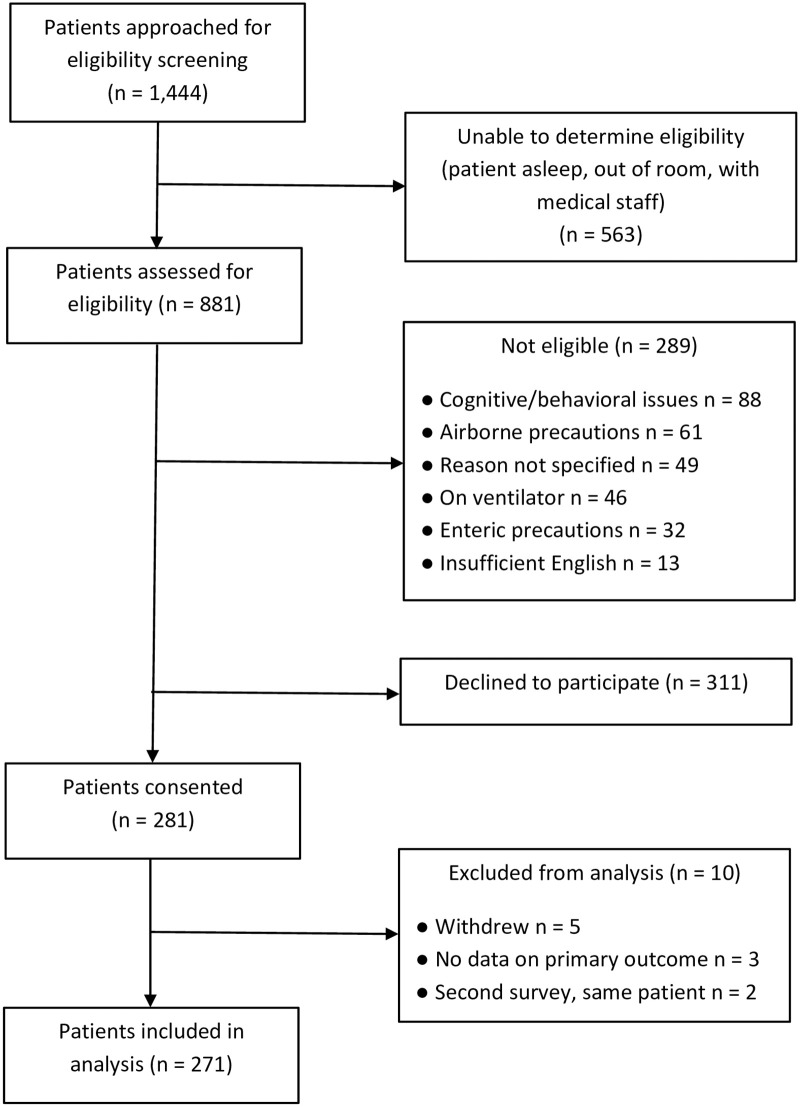
Participant recruitment process.

Fig 1 depicts the study sample recruitment. Of 1,444 inpatient rooms approached for participant recruitment, 276 patients were enrolled in the study. Five records were excluded from analysis, three because the participant had not completed the outcome measure for anxiety, and two that were inadvertent second interviews with patients who had already completed an initial survey. This left 271 patients in the study sample available for analysis.

In 563 rooms, patient eligibility could not be determined because the patient was asleep, medical staff were with the patient, or the patient was temporarily out of the room. In 289 rooms for which the patient was determined to be ineligible, they were excluded due to cognitive issues (30%), airborne precautions (21%), for unknown reasons (17%), patient on mechanical ventilation (16%), enteric precautions (11%), or due to the patient not speaking English (5%). In 311 rooms, the patient was eligible but declined to participate.

### Measures

#### Anxiety

We measured anxiety using the 6-item State-Trait Anxiety Inventory (STAI) and, in accordance with common practice, we prorated scores to a 20–80 range and defined clinically relevant anxiety as a STAI score ≥ 40 [24–26]. The 6-item STAI instrument has been validated against the 20-question state subscale of the STAI [27–29], which has been used extensively to measure anxiety in a variety of patient types [30–33]. A license to use the STAI was purchased from Mind Garden, Inc.

#### Stressors

We measured patient endorsement of 38 potential stressors such as pain, frustration, loss of control, inadequate social support, and shame (the complete list of stressors can be found in Table 2). Stressors were selected from the following sources: (1) from previous studies of distress among hospitalized patients [34–36], (2) from previous studies identifying stressors associated with anxiety in specific patient populations [5, 7–9, 14–16], (3) based on a review of spiritual assessment documentation tools currently in use in spiritual health departments of several top-rated hospitals in the U.S. (Northwestern Memorial Hospital, Chicago, Illinois; UCLA Health, Los Angeles, California; Duke University Hospital, Durham, North Carolina; Children’s Hospital, Greenville Health System, Greenville, South Carolina; Wexner Medical Center, Columbus, Ohio; Froedtert Hospital, Wauwatosa, Wisconsin; and Emory University Hospital, Atlanta, Georgia), and (4) based on the lead author’s experience delivering social and emotional care to inpatients as a spiritual health provider. To ensure that participants’ endorsement reflected stressors that were current and non-trivial, we introduced and asked the question as follows: “The goal of my visit today is to better understand things that may be burdening you or weighing on your heart while you have been in the hospital. … I will read you a list of concerns that other people in the hospital sometimes experience and please tell me, for each one, whether it is something that is a burden to you during this hospitalization.” Stressor endorsement was recorded as yes or no.

#### Demographics

Age, sex, and race were self-reported by participants. Insurance status was abstracted from the patient’s electronic medical record by a trained researcher. A second researcher reviewed 10% of the patient medical records to confirm abstracted data. No errors were identified.

### Statistical analysis

For our first aim, we used descriptive statistics (mean and standard deviation for continuous variables, frequency and percent for categorical variables) to characterize our study sample and to summarize the prevalence and distribution of stressors across hospital unit types. Chi-square tests were used to identify significant differences in stressor endorsement across demographic categories and unit type. ANOVA was used to identify associations between STAI score or number of stressors endorsed and unit type.

For our second aim, we performed a factor analysis to identify groups of stressors that tend to be endorsed together, excluding any stressor endorsed by fewer than 10% of participants as the limited variability could hinder the data decomposition function of the factor analysis. We used varimax rotation, which does not allow for correlation between stressors but resulted in stronger individual item loadings and the same factor identification compared to oblique rotation. We performed a two-step procedure modified from Raubenheimer to improve the reliability and validity of our factors [37]. First, we improved reliability by removing single variables iteratively from each factor to improve Cronbach’s alpha until no further improvements could be made. Second, validity was improved by removing stressors that did not load strongly and cleanly on a single factor. Items were removed if they had less than 0.40 loading on their primary factor and less than a 0.25 difference in loading between the primary and secondary factors. These thresholds were based on the procedure reported by Raubenheimer, although because we were not evaluating scales intentionally designed to measure mutually exclusive constructs but rather were seeking to investigate how stressors grouped together in patient experience, we modified the procedure to require loadings on the secondary factor to be at least 0.25 different from the primary factor instead of less than 0.25 [37].

To address our third aim, we performed two-tailed t-tests to compare mean STAI score for patients who endorsed each stressor with those who did not endorse that stressor to identify significant associations between anxiety and each of the 38 stressors. The Mann-Whitley U test was used for stressors not normally distributed and endorsed n < 30. Next, we used multivariate logistic regression to test for significant predictors of clinically relevant anxiety (STAI score ≥ 40). We ran four regression models: all 38 stressors, both with and without controlling for demographics (Models I and II) and the identified factors plus the remaining individual stressors, both with and without controlling for demographics (Models III and IV). Age was dichotomized at the approximate mean, as has been done in other studies examining association between anxiety and stressors [38]. Where data were missing for no more than one of the six items in the STAI scale, the missing value was imputed using the mean value of the remaining items. Three participants for which all six values were missing were not included in the analysis. All analyses were conducted using SAS 9.4 (The SAS Institute, Cary, NC) statistical analysis software. Statistical significance was set at an alpha level of 0.05.

## Results

### Description of study participants

Demographic characteristics of study participants and frequency by unit type are presented in Table 1. Mean age was 53.6 (standard deviation [SD] = 16.2) years. About half the participants were female (52.0%) and the sample had similar proportions of Black (48.2%) and White (45.6%) participants; only 6.2% reported another racial identity. Almost all participants were insured, either through private insurance (41.5%), Medicare (38.9%), or Medicaid (14.2%); only 5.4% were uninsured. Surgical services was the most represented unit type in our sample (19.6%), followed by hematology/oncology (16.6%), cardiovascular (14.4%), and cardiology (10.7%). The number of patients recruited from each unit type was roughly proportional to the number of beds in that unit type, with modest overrepresentation of cardiovascular and hematology/oncology units, and modest underrepresentation in general medicine, surgical, and bone marrow transplant units (S1 Table).

**Table 1:** Selected characteristics of hospital inpatient study participants (n = 271).

Characteristics	n or Mean^a^	% or SD ^a^
Age (Mean, SD)	53.6	16.2
Sex Female (n, %)	140	52.0
Race^b^ (n, %)		
White	88	45.6
African American/Black	93	48.2
Other	12	6.2
Insurance Status (n, %)		
None	14	5.4
Medicaid	37	14.2
Medicare	101	38.9
Private	108	41.5
Medical unit type (n, %)		
General medicine	23	8.5
Neurology	22	8.1
Cardiology	29	10.7
Cardiovascular	39	14.4
Pulmonary/Respiratory	7	2.6
Vascular	11	4.1
Solid organ transplant	19	7.0
Surgical services	53	19.6
Hematology/oncology	45	16.6
Bone marrow transplant	10	3.7
Renal/nephrology	13	4.8

^a^Mean and SD for numerical variables; percent and frequency (N) for categorical variables

^b^Race added late to survey, 29% missing; all other variables <5% missing

### Frequency and distribution of stressors

The proportion of stressor endorsement in the total sample and across unit types is presented in S2 Table. Overall, stressors were endorsed at levels ranging from 4.4% for marital troubles and anger at God/Higher Power, to 55.0% for pain. Following pain, the most highly endorsed items were feeling frustrated (endorsed by 49.5% of participants), inability to sleep (46.1%), feeling overwhelmed (43.5%), fear of the unknown about diagnosis and treatment (42.4%), loss of physical ability or bodily function (42.4%), missing out on important events in life (41.3%), and worry about quality of life (40.8%). These stressors are among the most frequently endorsed regardless of unit type, for example pain was among the top three endorsed stressors in all unit types. Feeling overwhelmed, feeling frustrated, fear of the unknown about diagnosis and treatment, and missing out on important life events were among the top ten endorsed stressors in over 90% of unit types. Inability to sleep and worry about quality of life were among the top ten endorsed stressors in at least 80% of unit types.

Despite these similarities in most frequently named stressors, some differences across unit types were noted. A loss of physical function and difficulty accepting one’s appearance were more frequently endorsed in neurology compared to other units (χ^2^: 25.45, p = .005; χ^2^: 22.69, p = .012 respectively). Inadequate support from family and regret were more frequently endorsed in the pulmonary unit (χ^2^: 18.54, p = .047; χ^2^: 21.45, p = .018 respectively). Anger at God was more frequently endorsed in the renal/nephrology units (χ^2^: 20.79, p = .023). There were no significant associations between unit type and either STAI score or number of stressors endorsed.

Table 2 presents bivariate associations between stressors and demographics. Compared to older patients, younger patients (≤ 55 years) more frequently endorsed pain (60.6% vs. 48.4%; χ^2^ = 3.98; p = .046), feeling frustrated (58.5% vs. 40.5%; χ^2^ = 8.63; p = .003), inability to sleep (52.8% vs. 39.7%; χ^2^ = 4.63; p = .032), feeling overwhelmed (49.3% vs. 37.3%; χ^2^ = 3.90; p = .048), and fear of death (35.2 vs. 15.9%; χ^2^ = 12.94; p = .0003). Women more frequently endorsed feeling overwhelmed (49.6% of women vs. 37.2% of men; χ^2^ = 4.20; p = .040), financial stress (40.3% vs. 28.7%; χ^2^ = 3.98; p = .046), loneliness (32.4% vs. 20.9%; χ^2^ = 4.46; p = .035), other family members ill or in trouble (29.5% vs. 16.3%; χ^2^ = 6.57; p = .010), difficulty accepting how they appear to others because of their illness (26.6% vs. 14.7%; χ^2^ = 5.72; p = .017), and feeling that others will or are judging them (20.1% vs. 10.9%; χ^2^ = 4.37; p = .037). Frequency of endorsement significantly differed by race for two stressors: difficult to be away from pets, which was more often endorsed by White patients (46.6% of White patients, 9.7% of Black patients, and 8.3% of other races; p = < .0001, Fisher’s exact test [FET]) and struggling with disconnection from Higher Power, which was more often endorsed by non-White patients (4.6% of White patients, 14.0% of Black patients, and 33.3% of other; p = .0005, FET). Frequency of endorsement significantly differed by type of health insurance for financial stress (50.0% of those with no insurance, 46.0% with Medicaid, 23.8% with Medicare, and 38.9% with private insurance; χ^2^ = 9.69; p = .021), fear of upcoming procedures (14.3%, 54.1%, 28.7%, and 31.5% respectively; p = .018, FET), loneliness (14.3%, 46.0%, 25.7%, and 20.4%; p = .020, FET), and conflicts with hospital staff (35.7%, 16.2%, 18.8%, and 7.4%; χ^2^ = 10.99; p = .012). Anxiety as measured by STAI score was higher for women compared to men (39.5 vs 35.0; t = 2.35; p = .019), and by patients insured under Medicaid (44.8) compared to Medicare (36.5), no insurance (36.2), or private insurance (34.7) (df = 3; F = 3.89; p = .010).

**Table 2:** Frequency of stressor endorsement across demographic characteristics.

Stressor	Percent Endorsing by Demographic^a^ (n)										
	Age		Sex		Race			Type of Health Insurance			
	≤ 55 (141)	> 55 (130)	Fem.(139)	Male (129)	White (88)	Black (93)	Other (12)	None (14)	M-A (101)	M-C (108)	Priv. (37)
Pain	**60.6**	**48.4** *	59.0	49.6	56.8	60.2	50.0	64.3	64.9	55.5	47.2
Feeling frustrated	**58.5**	**40.5** **	51.8	48.1	60.2	51.6	41.7	57.1	62.2	40.6	50.9
Inability to sleep	**52.8**	**39.7** *	48.2	44.2	50.0	50.5	66.7	64.3	51.4	39.6	47.2
Feeling overwhelmed	**49.3**	**37.3** *	**49.6**	**37.2** *	44.3	51.6	33.3	28.6	54.1	38.6	43.5
Fear of the unknown	47.2	38.1	46.0	39.5	53.4	41.9	41.7	50.0	51.4	36.6	42.6
Loss of physical ability/function	45.8	38.1	41.7	44.2	46.6	44.1	58.3	57.1	43.2	40.6	38.9
Missing out on important events	45.1	38.1	41.7	41.1	46.6	45.2	58.3	42.9	48.7	37.6	40.7
Worried about my quality of life	43.7	36.5	38.9	42.6	47.7	41.9	33.3	28.6	43.2	33.7	47.2
Feeling like I’ve lost control	38.0	35.7	41.0	32.6	42.1	33.3	41.7	35.7	37.8	34.7	38.0
Guilt over being a burden	38.7	33.3	39.6	32.6	46.6	36.6	25.0	21.4	40.5	28.7	42.6
Financial stress	39.4	28.6	**40.3**	**28.7** *	33.0	40.9	25.0	**50.0**	**46.0**	**23.8**	**38.9** *
Who will take care of my family	34.5	32.5	30.9	35.7	30.7	35.5	41.7	35.7	32.4	30.7	35.2
Fear of upcoming procedures	36.6	28.6	36.7	28.7	38.6	38.7	16.7	**14.3**	**54.1**	**28.7**	**31.5** *
Feeling discouraged	33.8	27.8	33.1	28.7	42.1	26.9	33.3	28.6	24.3	28.7	33.3
Disconnected from family/friends	31.7	25.4	28.8	27.1	34.1	28.0	33.3	21.4	35.1	28.7	25.9
Loneliness	28.9	23.8	**32.4**	**20.9** *	26.1	31.2	33.3	**14.3**	**46.0**	**25.7**	**20.4** *
Fear of death	**35.2**	**15.9** ***	30.9	20.9	28.4	32.3	25.0	21.4	37.8	24.8	24.1
Difficult to be away from pets	25.4	25.4	24.5	26.4	**46.6**	**9.7**	**8.3** ***	28.6	13.5	28.7	24.1
Worried about post-discharge care	26.1	23.0	25.9	23.3	25.0	31.2	16.7	14.3	37.8	17.8	26.9
Family members ill or in trouble	19.7	27.0	**29.5**	**16.3** *	30.7	18.3	25.0	14.3	27.0	18.8	25.9
Difficulty accepting how I appear	23.9	16.7	**26.6**	**14.7** *	20.5	28.0	16.7	7.1	37.8	15.8	19.4
Feelings of regret	17.6	21.4	20.1	18.6	19.3	25.8	16.7	14.3	18.9	22.8	18.5
No one to talk to about my illness	15.5	15.9	17.3	14.7	17.1	18.3	25.0	7.1	16.2	19.8	13.0
Feeling judged by others	19.7	11.1	**20.1**	**10.9** *	18.2	18.3	0.0	7.1	29.7	12.9	13.9
Conflicts with hospital staff	14.8	15.1	15.1	14.0	19.3	14.0	25.0	**35.7**	**16.2**	**18.8**	**7.4** *
Feeling hopeless	14.8	13.5	15.1	13.2	17.1	12.9	25.0	14.3	18.9	12.9	11.1
Feelings of low self-worth	14.1	13.5	15.1	12.4	15.9	12.9	0.0	7.1	8.1	17.8	11.1
Sense of guilt or shame	15.5	11.9	16.6	10.9	15.9	12.9	0.0	7.1	13.5	13.9	11.1
Need for forgiveness	13.4	12.7	14.4	11.6	13.6	16.1	0.0	14.3	21.6	10.9	10.2
My suffering is meaningless	11.3	12.7	15.8	8.5	11.4	12.9	25.0	14.3	10.8	15.8	7.4
Inadequate support from family	9.9	12.7	11.5	10.1	9.1	15.1	16.7	7.1	18.9	10.9	6.5
Loss of meaning or purpose in life	10.6	10.3	14.4	7.0	12.5	10.8	16.7	7.1	18.9	8.9	9.3
Disconnection from Higher Power	11.3	10.3	13.7	7.8	**4.6**	**14.0**	**33.3** **	7.1	10.8	11.9	9.3
Concerns about the afterlife	10.6	7.1	10.1	7.8	10.2	11.8	0.0	7.1	10.8	6.9	11.1
Questioning my faith	7.0	5.6	7.9	4.7	9.1	7.5	0.0	0.0	10.8	5.0	6.5
Feel abandoned/punished by God	7.0	3.2	2.9	7.8	2.3	8.6	8.3	7.1	10.8	5.0	3.7
Anger at God/Higher Power	3.5	5.6	5.0	3.9	4.6	3.2	0.0	7.1	8.1	5.0	2.8
Marital troubles	4.9	4.0	5.0	3.9	5.7	3.2	8.3	14.3	5.4	5.0	2.8
Anxiety (STAI Score)	38.9	35.7	**39.5**	**35.0** *	39.7	38.5	38.3	**36.2**	**44.8**	**36.5**	**34.7** **

^a^Fem. = female; M-A = Medicaid; M-C = Medicare; Priv. = private; age is dichotomized at the approximate mean

* p ≤ .05;

** p ≤ .01;

*** p ≤ .001

### Factor analysis

The factor analysis revealed two clusters of stressors that tended to be endorsed together: one relating to isolation and meaninglessness, and one relating to fear and frustration (Table 3). Although there were nine eigenvalues above 1 and the scree plot had a notable elbow at the third factor, running the factor analysis with three or more factors resulted, after factor optimization, with the additional “factors” having only a single stressor. Thus, the two-factor solution was retained.

**Table 3:** Result of factor analysis and optimization.

Stressor^a^^,^^b^	Loading^c^	
	Factor1	Factor2
No one to talk to about what I’m going through	**.81**	.24
Sense of guilt or shame	**.80**	.31
Feelings that I’ve lost meaning or purpose in life	**.80**	.32
Feeling that my suffering is meaningless	**.75**	.09
Loneliness	**.72**	.30
Feeling disconnected from my family, friends, communities of support	**.68**	.25
Feelings of low self-worth	**.67**	.40
Feeling hopeless	.66	.43
Inadequate support from family	**.62**	.21
Struggling with disconnection from Higher Power	**.59**	.12
Feeling like I’ve lost control	.57	.45
Feeling discouraged	.54	.54
Difficulty accepting how I appear toward others because of my illness	.53	.34
Missing out on important events in life	.53	.47
Need for forgiveness	.53	.30
Feeling that others will or are judging me	.51	.36
Other family members ill or in trouble	.45	.31
Loss of physical ability or bodily function	.40	.38
Feelings of regret	.39	.36
Fear of the unknown about diagnosis and treatment	.07	**.86**
Fear of upcoming procedures	.08	**.70**
Fear of death	.18	**.69**
Feeling frustrated	.40	**.67**
Feeling overwhelmed	.39	.63
Worried about my quality of life	.33	**.59**
Worried about who will take care of me	.42	.57
Guilt over being a "burden" to family members	.41	.51
Inability to sleep	.26	.44
Financial stress	.30	.39
Worried about who will take care of my family if I can’t	.31	.32
Number of stressors in optimized factor	9	4
Cronbach’s alpha of optimized factor	.81	.72

^a^Stressors endorsed < 10% were not included in the factor analysis (feeling abandoned by God/Higher Power, questioning my faith, anger at God/Higher Power, concerns about the afterlife, marital troubles)

^b^Three additional stressors (pain, conflicts with staff, and difficult to be away from pets) do not appear in the table because, in accordance with the factor optimization procedure modified from Raubenheimer (2004), they were removed to increase factor reliability (based on Cronbach’s alpha) before optimization of factor validity

^c^Stressors with individual loadings ≥ .40 and at least .25 difference between primary and secondary factors were retained; retained items shown in bold

An isolation and meaninglessness factor includes nine stressors with factor loadings between 0.59 and 0.81: having no one to talk to, a sense of guilt or shame, loss of meaning or purpose in life, feeling that suffering is meaningless, loneliness, feeling disconnected from family/friends/communities of support, feelings of low self-worth, inadequate support from family, and struggling with disconnection from a Higher Power (Cronbach’s alpha = 0.81). A fear and frustration factor includes five stressors with factor loadings between 0.59 and 0.86: fear of the unknown about diagnosis and treatment, fear of upcoming procedures, fear of death, worry about quality of life, and feeling frustrated (Cronbach’s alpha = 0.72). All remaining stressors lacked strong loading on a single factor, and therefore may be considered to travel independently rather than in a patterned group with other stressors.

### Stressor association with anxiety

Based on t-test comparison of mean STAI scores between those who did and did not endorse each stressor, we found that each of the 38 stressors was significantly associated with anxiety: p < .0001 for 29 stressors, p < .001 for five stressors, p < .01 for three, p = .043 for a single stressor (feeling abandoned or punished by God) (S3 Table). Next, we conducted multivariable logistic regression, first in Model I with only the 38 stressors, and then in Model II controlling for demographic variables including age, sex, race, and insurance status (Table 4). In Model I, stressors significantly associated with clinically relevant anxiety include pain (odds ratio [OR] = 2.27; 95% confidence interval [CI] = [1.02, 5.06]; p = .045), feeling frustrated (OR = 3.34; CI = [1.41, 7.94]; p = .006), loss of control (OR = 2.98; CI = [1.19, 7.45]; p = .020), financial stress (OR = 2.20; CI = [1.00, 4.83]; p = .049), and loneliness (OR = 2.9; CI = [1.07, 7.84]; p = .036).

**Table 4:** Logistic regression of clinically relevant anxiety^a^ on stressors (Model I) and adjusted for demographics (Model II).

Parameter	Model I				Model II			
	n = 271				n = 266^a^			
	OR	95% CI		p	OR	95% CI		p ^b^
Pain	2.27	1.02	5.06	**0.045**	2.34	1.02	5.35	**0.044**
Feeling frustrated	3.34	1.41	7.94	**0.006**	3.72	1.52	9.15	**0.004**
Inability to sleep	0.77	0.35	1.70	0.511	0.84	0.36	1.93	0.674
Feeling overwhelmed	1.19	0.52	2.75	0.680	1.07	0.44	2.61	0.889
Fear of the unknown about diagnosis and treatment	1.48	0.61	3.57	0.382	1.39	0.55	3.52	0.491
Loss of physical ability or bodily function	1.34	0.60	2.95	0.475	1.50	0.64	3.49	0.350
Missing out on important events in life	0.65	0.26	1.64	0.361	0.77	0.30	2.01	0.595
Worried about my quality of life	1.68	0.68	4.13	0.261	1.51	0.57	3.98	0.407
Feeling like I’ve lost control	2.98	1.19	7.45	**0.020**	2.81	1.09	7.29	**0.033**
Guilt over being a "burden" to family members	0.85	0.35	2.06	0.724	0.88	0.36	2.18	0.786
Financial stress	2.20	1.00	4.83	**0.049**	2.24	0.97	5.18	0.058
Worried about who will take care of my family if I can’t	0.80	0.32	2.00	0.626	0.84	0.32	2.17	0.714
Fear of upcoming procedures	1.84	0.73	4.60	0.194	1.81	0.69	4.77	0.231
Feeling discouraged	0.87	0.33	2.32	0.778	0.97	0.35	2.71	0.954
Feeling disconnected from my family, friends, communities of support	1.06	0.37	3.03	0.915	1.10	0.36	3.36	0.872
Loneliness	2.90	1.07	7.84	**0.036**	2.59	0.90	7.45	0.077
Fear of death	0.85	0.33	2.23	0.745	0.88	0.33	2.38	0.800
Difficult to be away from pets	0.56	0.22	1.44	0.230	0.54	0.20	1.48	0.232
Worried about who will take care of me	1.29	0.49	3.43	0.610	1.50	0.54	4.16	0.434
Other family members ill or in trouble	0.84	0.32	2.24	0.732	0.71	0.25	2.03	0.523
Difficulty accepting how I appear toward others because of my illness	1.00	0.36	2.75	0.999	1.16	0.39	3.42	0.789
Feelings of regret	1.22	0.44	3.39	0.710	1.18	0.40	3.44	0.765
No one to talk to about what I’m going through	0.53	0.13	2.21	0.380	0.63	0.14	2.79	0.539
Feeling that others will or are judging me	0.74	0.21	2.55	0.630	0.58	0.15	2.21	0.425
Conflicts with hospital staff	2.08	0.70	6.25	0.190	1.92	0.59	6.30	0.282
Feeling hopeless	3.99	0.87	18.23	0.074	4.37	0.86	22.27	0.076
Feelings of low self-worth	1.21	0.29	5.09	0.791	1.37	0.31	6.10	0.684
Sense of guilt or shame	3.17	0.57	17.68	0.189	3.65	0.60	22.36	0.161
Need for forgiveness	1.20	0.29	4.92	0.800	0.98	0.22	4.39	0.983
Feeling that my suffering is meaningless	1.40	0.32	6.05	0.652	1.63	0.34	7.68	0.539
Inadequate support from family	1.83	0.42	7.94	0.422	2.01	0.44	9.15	0.369
Feelings that I’ve lost meaning or purpose in life	0.63	0.11	3.62	0.605	0.52	0.08	3.64	0.513
Struggling with disconnection from Higher Power	0.95	0.24	3.80	0.944	0.65	0.15	2.80	0.565
Concerns about the afterlife	2.78	0.63	12.24	0.177	4.07	0.87	19.08	0.075
Questioning my faith	4.10	0.40	42.17	0.236	3.29	0.30	36.40	0.332
Feeling abandoned or punished by God	0.18	0.02	1.42	0.105	0.22	0.03	1.84	0.163
Anger at God/Higher Power	8.09	0.43	152.7	0.163	6.72	0.35	127.5	0.205
Marital Troubles	1.42	0.15	13.61	0.763	1.19	0.11	13.54	0.887
Age					1.01	0.98	1.04	0.465
Male sex (reference = female)					0.68	0.31	1.51	0.346
Race^a^ (reference = white)								
Black					0.59	0.24	1.44	0.243
Other					1.21	0.17	8.54	0.846
Insurance status (reference = no insurance)								
Medicaid					2.05	0.38	11.06	0.405
Medicare					0.91	0.20	4.06	0.901
Private					0.79	0.19	3.39	0.752

^a^ Clinically relevant anxiety is defined by a STAI score ≥ 40

^b^ Race added late to survey, 29% missing

^c^ Statistical significance set at p ≤ .05, bolded

After controlling for demographics, the stressors significantly associated with anxiety include pain (OR = 2.34; CI = [1.02, 5.35]; p = .044), feeling frustrated (OR = 3.72; CI = [1.52, 9.15]; p = .004), and loss of control (OR = 2.81; CI = [1.09, 7.29]; p = .033). Although anxiety was significantly associated with financial stress and loneliness in the unadjusted model, once adjusted these associations become non-significant, although both have a p-value at the trend level (p = .058 and p = .077, respectively). None of the demographic variables are significantly associated with anxiety in model II.

Models III and IV include the two multi-stressor factors as well as the remaining separate stressors (Table 5). In Model III, stressors significantly associated with anxiety include the isolation/meaninglessness factor (OR = 1.30; CI = [1.01, 1.68]; p = .043), the fear/frustration factor (OR = 1.62; CI = [1.18, 2.21]; p = .003), pain (OR = 2.09; CI = [1.00, 4.36]; p = .049), loss of control (OR = 2.78; CI = [1.21, 6.41]; p = .016), financial stress (OR = 2.17; CI = [1.02, 4.61]; p = .044), and feeling hopeless (OR = 4.04; CI = [1.04, 15.67]; p = .044). After controlling for demographics (Model IV), the stressors significantly associated with anxiety include the isolation/meaninglessness factor (OR = 1.32; CI = [1.01, 1.74]; p = .046), the fear/frustration factor (OR = 1.60; CI = [1.15, 2.23]; p = .005), and loss of control (OR = 2.70; CI = [1.14, 6.41]; p = .024). Although anxiety was significantly associated with pain, financial stress and feeling hopeless in the unadjusted model, once adjusted these associations become non-significant, although all have a p-value at the trend level (p = .060, p = .066, and p = .065, respectively). None of the demographic variables are significantly associated with anxiety in Model IV.

**Table 5:** Logistic regression of clinically relevant anxiety^a^ on factors and stressors (Model III) and adjusted for demographics (Model IV).

Parameter	Model I				Model II			
	n = 271				n = 266^a^			
	OR	95% CI		p	OR	95% CI		p ^b^
Isolation/meaninglessness factor	1.30	1.01	1.68	**0.043**	1.32	1.01	1.74	**0.046**
Fear/frustration factor	1.62	1.18	2.21	**0.003**	1.60	1.15	2.23	**0.005**
Pain	2.09	1.00	4.36	**0.049**	2.06	0.97	4.37	0.060
Inability to sleep	0.85	0.41	1.77	0.667	0.93	0.44	1.99	0.860
Feeling overwhelmed	1.23	0.56	2.72	0.609	1.08	0.47	2.51	0.859
Loss of physical ability or bodily function	1.16	0.55	2.45	0.699	1.24	0.56	2.74	0.594
Missing out on important events in life	0.64	0.27	1.52	0.313	0.70	0.29	1.70	0.432
Worried about my quality of life	0.93	0.39	2.24	0.873	0.85	0.33	2.19	0.735
Feeling like I’ve lost control	2.78	1.21	6.41	**0.016**	2.70	1.14	6.41	**0.024**
Guilt over being a "burden" to family members	1.13	0.51	2.52	0.766	1.17	0.51	2.68	0.719
Financial stress	2.17	1.02	4.61	**0.044**	2.09	0.95	4.58	0.066
Worried about who will take care of my family if I can’t	0.86	0.40	1.86	0.698	0.93	0.42	2.07	0.861
Feeling discouraged	1.04	0.43	2.52	0.940	1.23	0.49	3.13	0.659
Difficult to be away from pets	0.72	0.30	1.73	0.466	0.73	0.29	1.84	0.511
Worried about who will take care of me	1.24	0.49	3.15	0.644	1.42	0.54	3.71	0.480
Other family members ill or in trouble	0.88	0.35	2.23	0.793	0.84	0.32	2.22	0.723
Difficulty accepting how I appear toward others because of my illness	0.95	0.37	2.42	0.906	1.08	0.40	2.93	0.885
Feelings of regret	1.10	0.42	2.86	0.844	1.09	0.40	2.97	0.859
Feeling that others will or are judging me	0.77	0.25	2.37	0.652	0.69	0.22	2.20	0.530
Conflicts with hospital staff	1.91	0.69	5.28	0.216	1.76	0.60	5.20	0.305
Feeling hopeless	4.04	1.04	15.67	**0.044**	3.85	0.92	16.17	0.065
Need for forgiveness	1.25	0.39	4.04	0.707	1.01	0.30	3.37	0.989
Concerns about the afterlife	1.79	0.46	6.97	0.403	2.23	0.55	8.99	0.258
Questioning my faith	2.45	0.31	19.26	0.395	1.90	0.25	14.74	0.537
Feeling abandoned or punished by God	0.15	0.02	1.01	0.051	0.17	0.02	1.16	0.070
Anger at God/Higher Power	8.00	0.66	97.59	0.103	5.67	0.45	72.23	0.181
Marital Troubles	1.25	0.15	10.16	0.835	1.09	0.12	9.62	0.938
Age					1.01	0.98	1.04	0.547
Male sex (reference = female)					0.79	0.38	1.66	0.539
Race^a^ (reference = white)								
Black					0.72	0.31	1.66	0.442
Other					0.84	0.12	5.72	0.854
Insurance status (reference = no insurance)								
Medicaid					1.60	0.35	7.31	0.546
Medicare					0.69	0.18	2.67	0.593
Private					0.60	0.16	2.23	0.443

^a^ Clinically relevant anxiety is defined by a STAI score ≥ 40

^b^ Race added late to survey, 29% missing

^c^ Statistical significance set at p ≤ .05, bolded

## Discussion

Previous research indicates that anxiety is highly prevalent among hospital inpatients, regardless of morbidity, and robust clinical and epidemiological evidence suggests that anxiety, when comorbid with other medical conditions, exerts harmful effects [2, 3, 10, 39–42]. Here, we found that a subset of stressors including pain, being unable to sleep, feelings of frustration, being overwhelmed, and fear of the unknown were highly endorsed across all unit types. Factor analysis of patient responses produced two multi-item factors relating to isolation/meaningless and fear/frustration factors. Younger patients and women more frequently endorsed a number of stressors, as did patients without health insurance or insured under Medicaid. In bivariate analysis, all stressors were associated with anxiety; however, once included in multivariate modeling, only pain, frustration, financial distress, loneliness, and loss of control remained significantly associated with anxiety. After accounting for the sources of distress, patient age, gender, race, and insurance status no longer predicted anxiety, indicating that these stressors may mediate the relationship between these sociodemographic factors and increased risk for anxiety.

Post-discharge mental health has profound importance for long-term patient outcomes in terms of cost [43] and readmissions rates [44], and so adequately identifying and addressing mental health needs of hospital inpatients is critical. While developing and adapting interventions for specific patient populations is crucial, and a handful of randomized control trials examining interventions to reduce anxiety have been completed in targeted outpatient populations [5], there are some scenarios in which the ability to tailor an intervention for the hospital experience writ large is necessary, primarily to address common concerns among patients across disease types and treatment units, both for greater inclusion of those in need of an intervention and simplicity of having a single (or few) interventions to deploy. Similarly, there are hospital workforce staff who work across units (for example, hospital chaplains) and for whom a more general and less siloed understanding of hospital distress is important. Moreover, although anxiety is common among inpatients, there are a paucity of options available to target subclinical levels of anxiety that may not meet criteria for psychiatric intervention.

### Aim 1: Stressor prevalence, distribution, and crude association with anxiety

Our findings were consistent with other quantitative studies that found high endorsement of pain [45, 46], insomnia and sleep disruption [47], and fear and low endorsement of religious struggle. However, compared to others we found substantially lower endorsement of struggles around the meaning of suffering and meaning and purpose in life, perhaps due to cultural or religious differences between our U.S. population and the comparative study populations in Italy and Portugal [2, 48]. Our study also showed greater prevalence of stressors compared to qualitative studies that tallied stressor mentions; our data likely reflect participants’ more frequent recall of stressors when responding to survey prompts compared to more open-ended inquiries [49].

The most commonly-endorsed stressors were relatively consistent across unit types and included pain, feeling frustrated, inability to sleep, feeling overwhelmed, fear of the unknown about diagnosis and treatment, loss of physical ability and bodily function, missing out on important events in life, and worry about quality of life. These data suggest that hospital-wide interventions and generalized approaches to address these stressors could be effective regardless of disease type. Such broadly-applied interventional approaches have been shown to be effective in other studies. For example, in a study of 244 hospital inpatients, a brief mind-body intervention (mindfulness or hypnotic suggestion) delivered to a multi-morbid and heterogeneous population was found to reduce pain (23% reduction in pain with mindfulness and 29% with suggestion, compared to 9% with psychoeducation). A statistically significant reduction in anxiety was reported for all three arms of the study, although the effect size was not provided [17].

Despite this relative concordance across unit types, we did observe some notable differences. Neurology inpatients reported greater struggle with physical function and appearance compared to other units, while those on pulmonary/respiratory units reported inadequate family support and regret. Renal/nephrology inpatients more frequently reported being angry at God, although this stressor had overall low endorsement. These results should be viewed with caution based on the low endorsement of some stressors and the relatively small sample sizes, especially on the pulmonology unit which provided only seven study participants (Table 1). However, our findings appear consistent with the literature. For example, others have found living alone and social disengagement to be risk factors for hospitalization with respiratory disease [50], which aligns with the lack of family support we identified. Further, anger has been identified as a common response to kidney disease [51, 52], and negative religious coping such as anger at God has been found to be present in nephrology patients and associated with poorer mental health and social functioning [53].

Stressors were generally more prevalent among younger, female, and uninsured or Medicaid-insured patients. Some of these statistically-significant differences across demographics are broadly consistent with previous research. For example, women, who are often responsible for caring for others and who are socialized to find value in physical appearance [54–56], were more likely to report struggle with other family members being ill or in trouble, difficulty accepting how they appear because of their illness, and feeling judged. Previous research has found that women hospitalized for emergency surgery had higher levels of anxiety [7]. Younger participants were more likely to report feeling distressed by pain, frustration, inability to sleep, feeling overwhelmed, fear of death, and feeling judged, perhaps reflecting the development that comes with age of psychological resources to preserve well-being in spite of serious medical illness [57, 58]. Interestingly, there was not a significant effect of age on anxiety levels. The stressor with the greatest difference across groups was missing a pet; nearly half of White patients endorsed this as a difficulty compared to few Black patients or patients of other races. This may reflect a cultural difference in attitudes toward pets and/or a difference in pet ownership rates. Older adults who identify as Black have been shown to have lower rates of pet ownership and pet bonding compared to those identifying as White [59]. People with no insurance most often reported conflicts with staff, by a wide margin, perhaps driven by health provider bias against patients with lower socioeconomic status and patients’ perceptions of unequal care [60, 61].

### Aim 2: Stressor groupings

The results of our factor analysis suggest thematic groupings of stressors into categories of isolation/meaninglessness and fear/frustration. To our knowledge, this is the first study to utilize factor analysis to group inpatient stressors, although other studies have used qualitative methods to thematically group stressors expressed by patients. For example, Feuchtinger and colleagues grouped patients’ fears and anxieties into broad categories of fears, negation of fears, and other emotional and physical conditions, the latter of which included items as diverse as positive emotions, negative emotions, sleep problems, and pain [49]. An analysis of palliative care patient responses to the question “What bothers you the most?” shows this same tendency to group concerns in broad thematic categories, two of which are particularly broad: “emotional, spiritual, existential, or nonspecific distress,” and “relationships” [62]. In a meta-analysis on distress expressed by lung cancer patients, Refsgaard and Frederickson identified eight themes: guilt, blame, shame, and stigmatization; hope and despair; loneliness; change in self-image and self-worth; uselessness and dependency; uncertainty and worries; anxiety and fear; and loss [63]. Our factor analysis indicates that stressors agglomerated by others qualitatively may not fall within the same thematic group, and our method of querying a diverse but distinct array of stressors coupled with factor analysis of patient endorsement yields new information about how stressors may relate. For example, our findings indicate that low self-worth is more related to feelings of disconnection and lost meaning than it is to appearance, as suggested by Refsgaard and Frederickson.

We identified a substantial number of individual stressors that did not robustly load onto any factor, even if they appeared on their face to be related to a common theme. For example, several stressors arguably relate to physical impairment: loss of physical ability or bodily function, worries about quality of life, worries about who will provide care after discharge, and guilt over being a burden to family members. However, these stressors did not load onto a single factor, indicating that they are not consistently endorsed together in our sample. Similarly, while we found a sense of guilt or shame and feelings of low self-worth to group together, the factor did not include other stressors that on their face might appear to be related, for example, feeling regret or feeling a need for forgiveness.

Taken together, the discrepancies between our findings and those from previous qualitative studies indicate that the degree to which stressors covary or appear independent could be obscured by a solely qualitative process for determining distress themes. The thematic analysis process for qualitative approaches tend to group items based on face validity only, without regard for the frequency with which they appear together in the data. This study contributes to the overall understanding of patient distress by quantitatively evaluating relationships among stressors.

### Aim 3: Stressor association with anxiety

Like others, we found multiple stressors to be significantly associated with anxiety in bivariate analyses [8, 12, 14]. However, once all stressors were included in a multivariate model, only five stressors remained significant predictors of anxiety: pain, frustration, feeling a loss of control, financial stress, and loneliness. We then controlled for demographics, which revealed three stressors that were associated with anxiety: pain, frustration, and loss of control. Models including multi-stressor factors yielded similar results: the two factors described as isolation/meaninglessness and fear/frustration were associated with anxiety, as well as pain, loss of control, financial stress, and feeling hopeless in the unadjusted model and pain and loss of control in the adjusted model. Predictors of anxiety in other studies were largely consistent with our results, for example pain, loss of control, isolation, and lack of meaning have been identified as associated with anxiety by others [8, 11, 12, 38]. The consistency of findings is striking, considering these studies generally included only a small subset of the stressors included in our model and others’ more narrowly targeted study populations, for example patients under palliative care or treatment for cancer, were not as wide ranging as our hospital-wide sample. This may be an indication of the commonality of stressors across disease types and their potential impact on wellbeing and points to the potential benefit of broad interventions.

It is noteworthy that previous studies have consistently identified female gender and intermittently identified younger age and indicators of socioeconomic status (education and income) to be associated with anxiety in a variety of domestic and international patients hospitalized with a range of acute and chronic ailments [10–13, 64, 65]. Although we identified female gender and insurance status (a proxy for socioeconomic status) to be associated with anxiety in bivariate analysis, demographics were not associated with anxiety in our multivariable models, which included many stressors related to existential and emotional struggles. This is a marked departure from others’ findings. Our findings suggest that the widely-identified association of anxiety with demographics, particularly female sex but also younger age and lower socio-economic status, may be mediated by these existential and emotional stressors.

Results of the current study add to what is known about hospital stressors associated with anxiety among hospitalized patients and provide a solid foundation for the development of targeted interventions to be delivered at the bedside. Previous research has highlighted the importance of whole systems approaches to address persistent problems in the hospital environment. For example, one scoping review concluded that sleep disturbance due to noise is best addressed with comprehensive and multi-level approaches that target both the hospital environment and staff and provider education and attitudes [66]. Our data support such an approach with the finding that several stressors and groups of stressors are related to anxiety and widely prevalent regardless of hospital unit.

### Strengths and limitations

Major strengths of this study include the quantitative approach and analytical rigor employed in determining thematic distress factors and evaluating the association between stressors and anxiety. The inclusion of patients from all inpatient units provides a more complete picture of anxiety among patients than is available from previous studies, many of which have been limited to specific patient populations such as cancer, palliative care, cardiac, or surgical patients. This fuller understanding of the nature and distribution of stressors within the hospital will assist both in developing interventions that can be used across a wide range of patient types and in deploying resources strategically to segments of the patient population or areas of the hospital carrying the most distress.

This study, like all cross-sectional studies, is limited to identifying associations between variables and cannot determine causality. Moreover, the inclusion of patients from a single hospital system in a single part of the country limits generalizability. The use of a binary yes/no option when asking patients if they were burdened by each stressor limited the degree of nuance we were able to capture. Our data would have been richer, and the degree of distress would have been more readily calculable, had we asked about each stressor on a Likert-type scale. Enabling a range of answers to each stressor question would have allowed the creation of a more nuanced score both of single stressors and within each identified factor, although this would also have added to patient burden in completing the survey.

In addition, the potential for selection bias is present in our study. Because we were not able to gather data on stressors, anxiety, or chart-abstracted demographics from patients who did not consent to the study, we were unable to evaluate whether those who participated were systematically different from those who did not. Patients who were in a great deal of pain may have been underrepresented in our study population, as these patients may have been unwilling or too cognitively impaired to participate due to medication status or distress levels. It is possible that the patients experiencing the most distress were those who were ineligible for recruitment due to being heavily medicated, sedated, or on a ventilator, or who were least likely to enroll when approached. Social desirability bias may have resulted in participants under- or over-reporting stressor endorsement. The prevalence of stressors and the estimates of the association between stressors and STAI score evident in these data may therefore be underestimated.

Importantly, this study examined the relationship between stressors and anxiety, but it remains to be seen how these same stressors may differentially predict other outcomes of interest such as depression. For example, previous studies have found that medical uncertainty experienced by hospitalized cardiac patients was associated with both anxiety and depression, and that pessimism, worthlessness, and loss of interest in others were associated with major depression in medically ill patients [67, 68]. This future research will be important, especially given the high prevalence of depression among hospital inpatients [69] and given research indicating that inpatient depression is associated with both length of stay and risk for readmission [70]. In addition, we are reminded of the importance of promoting psychological well-being and flourishing, even in the face of serious illness [71], and the data here are limited in their focus on anxiety.

### Conclusion

Several studies have identified physical, psychological, and social predictors of anxiety among discrete inpatient populations and can be used to inform a general understanding of inpatient anxiety. For example, anxiety has been found to be associated with insufficient sleep, pain, inadequate explanation of the treatment, separation from family, uncertainty, loss of control, fear, and impaired body image among pre- and post-surgical patients [5, 7–9]; and with physical symptoms, financial instability, social support or isolation, shame, and stress among cancer patients [15, 16]. We found these and other stressors widely distributed through our heterogeneous hospital-wide sample, with stressors relating to isolation, lack of meaning, fear, frustration, and lack of control being associated with anxiety. Our study adds to the existing body of evidence and provides important context for understanding the contours of inpatient distress and developing interventions to reduce distress and anxiety in the hospital. Further research is warranted to examine associations between hospital distress and other mental and emotional outcomes such as depression and wellbeing. There is also a need for longitudinal studies examining psychological, emotional, and spiritual care and their impact on these outcomes.

## Supporting information

S1 File Study survey. (DOCX)

S1 Table Recruitment of participants by unit type. (DOCX)

S2 Table Frequency of stressor endorsement across hospital unit type. (DOCX)

S3 Table Association between stressors and mean STAI score. (DOCX)
