# Supplementary material for: The prevalence, grouping, and distribution of stressors and their association with anxiety among hospitalized patients
Source: PLoS One. 2021 Dec 6;16(12):e0260921. doi: 10.1371/journal.pone.0260921 (PMC8648119; doi:10.1371/journal.pone.0260921)
Supplement: S1 File — (DOCX) [file pone.0260921.s001.docx]

**Supporting materials: Study Survey**

Questions and available answer options were read verbally to patient to reduce burden; responses were typed into an electronic pad by the researcher.

Age ____________________

Sex ____________________

Race:

⭘ American Indian/Alaskan Native

⭘ Native Hawaiian or Other Pacific Islander

⭘ White

⭘ African American/Black

⭘ Asian

⭘ Other

Ethnicity:

⭘ Hispanic or Latino

⭘ Not Hispanic or Latino

⭘ Unknown

Marital Status:

⭘ Single

⭘ Single, divorced

⭘ Single, but living with someone

⭘ In a relationship

⭘ In a relationship but living separately

⭘ Married

⭘ Widowed

Education: What is the highest degree or level of school you have completed? If you’re currently enrolled in school, please indicate the highest degree you have received.

⭘ Grade school

⭘ High school or equivalent

⭘ Some college, no degree

⭘ Associate degree (2 year)

⭘ Bachelor’s degree (e.g.

⭘ Master’s degree (e.g.

⭘ Professional degree (e.g.

⭘ Doctorate (e.g. PhD, Ed

The goal of my visit today is to better understand things that may be burdening you or weighing on your heart while you have been in the hospital. I will read a list of concerns that other people in the hospital sometimes experience and please tell me, for each one, whether it is something that is a burden to you during this hospitalization.

⭘ Pain

⭘ Regret

⭘ Feelings of low self-worth

⭘ Difficulty accepting how I appear to others because of my illness

⭘ Feeling overwhelmed

⭘ Feeling like I’ve lost control

⭘ Feeling disconnected from family, friends, communities of support

⭘ No one to talk to about what I’m going through

⭘ Feeling that my suffering is meaningless

⭘ Feeling that I’ve lost meaning or purpose in life

⭘ Loneliness

⭘ Difficult to be away from pet(s)

⭘ Feeling hopeless

⭘ Feeling frustrated

⭘ Feeling discouraged

⭘ Sense of guilt or shame

⭘ Feeling that others will or are judging me

⭘ Need for forgiveness

⭘ Fear of upcoming procedure

⭘ Fear of death

⭘ Fear of the unknown about diagnosis and treatment

⭘ Worried about my quality of life

⭘ Worried about who will take care of my family if I can’t

⭘ Worried about who will take care of me

⭘ Loss of physical ability or bodily function

⭘ Missing out on important events in life

⭘ Struggling with disconnection from Higher Power

⭘ Feeling abandoned or punished by God/Higher Power

⭘ Questioning my faith

⭘ Anger at God/Higher Power

⭘ Concerns about the afterlife

⭘ Conflicts the hospital staff

⭘ Guilt over being a “burden” to family members

⭘ Inadequate support from family

⭘ Other family members/friends ill or in trouble

⭘ Marital troubles

⭘ Inability to sleep

⭘ Financial stress

STAI-6

[Questions redacted]

The six-item short form of the Spielberger State-Trait Anxiety Inventory (STAI) was developed by Theresa M. Marteau and Hilary Bekker (1992) and was used in this study with a license from Mind Garden, Inc., [www.mindgarden.com](http://www.mindgarden.com). Reproduction from the State-Trait Anxiety Inventory for Adults by Charles D. Spielberger (Copyright © 1968, 1977 by Charles D. Spielberger) is prohibited without the Publisher’s written consent.
