# Supplementary material for: The prevalence, grouping, and distribution of stressors and their association with anxiety among hospitalized patients
Source: PLoS One. 2021 Dec 6;16(12):e0260921. doi: 10.1371/journal.pone.0260921 (PMC8648119; doi:10.1371/journal.pone.0260921)
Supplement: S1 Table — (DOCX) [file pone.0260921.s002.docx]

| **Unit Type** | **No. Beds** | **N** | **% Total Beds** | **% Total N** |
| --- | --- | --- | --- | --- |
| General medicine | 111 | 23 | 11.9 | 8.5 |
| Neurology | 77 | 22 | 8.3 | 8.1 |
| Cardiology | 80 | 29 | 9.8 | 10.7 |
| Cardiovascular | 91 | 39 | 8.6 | 14.4 |
| Pulmonary/respiratory | 18 | 7 | 1.9 | 2.6 |
| Vascular | 20 | 11 | 2.1 | 4.1 |
| Transplant | 69 | 19 | 7.4 | 7.0 |
| Surgical | 243 | 53 | 26.1 | 19.6 |
| Hematology/oncology | 111 | 45 | 11.9 | 16.6 |
| Bone marrow transplant | 64 | 10 | 6.9 | 3.7 |
| Renal/nephrology | 48 | 13 | 5.2 | 4.8 |
