# Supplementary material for: The prevalence, grouping, and distribution of stressors and their association with anxiety among hospitalized patients
Source: PLoS One. 2021 Dec 6;16(12):e0260921. doi: 10.1371/journal.pone.0260921 (PMC8648119; doi:10.1371/journal.pone.0260921)
Supplement: S2 Table — (DOCX) [file pone.0260921.s003.docx]

| **Stressor** | **Percent Endorsing by Unit Type (n)** | | | | | | | | | | | |
| --- | --- | --- | --- | --- | --- | --- | --- | --- | --- | --- | --- | --- |
|  | **Total** | **Med (23)** | **Neur (22)** | **Card (29)** | **CV (39)** | **Resp (7)** | **Vasc (11)** | **Tran (19)** | **Surg (53)** | **Onc (45)** | **BMT (10)** | **Ren (13)** |
| Pain | 55.0 | 56.5 | 59.1 | 51.7 | 51.3 | 71.4 | 45.5 | 47.4 | 52.8 | 55.6 | 50.0 | 84.6 |
| Feeling frustrated | 49.5 | 34.8 | 59.1 | 55.2 | 53.9 | 57.1 | 45.5 | 36.8 | 54.7 | 51.1 | 50.0 | 23.1 |
| Inability to sleep | 46.1 | 30.4 | 63.6 | 51.7 | 51.3 | 57.1 | 45.5 | 31.6 | 47.2 | 48.9 | 50.0 | 15.4 |
| Feeling overwhelmed | 43.5 | 39.1 | 54.6 | 37.9 | 43.6 | 57.1 | 27.3 | 36.8 | 50.9 | 44.4 | 40.0 | 30.8 |
| Fear of the unknown about diagnosis and treatment | 42.4 | 30.4 | 59.1 | 48.3 | 43.6 | 57.1 | 27.3 | 36.8 | 47.2 | 40.0 | 40.0 | 23.1 |
| **Loss of physical ability or bodily function**** | 42.4 | 26.1 | 77.3 | 37.9 | 46.2 | 42.9 | 9.1 | 36.8 | 54.7 | 35.6 | 20.0 | 38.5 |
| Missing out on important events | 41.3 | 26.1 | 40.9 | 37.9 | 38.5 | 42.9 | 36.4 | 47.4 | 49.1 | 40.0 | 60.0 | 38.5 |
| Worried about my quality of life | 40.6 | 30.4 | 45.5 | 51.7 | 35.9 | 42.9 | 36.4 | 42.1 | 47.2 | 31.1 | 50.0 | 38.5 |
| Feeling like I've lost control | 36.5 | 34.8 | 50.0 | 34.5 | 35.9 | 42.9 | 18.2 | 36.8 | 37.7 | 33.3 | 40.0 | 38.5 |
| Guilt over being a "burden" | 35.8 | 26.1 | 36.4 | 31.0 | 48.7 | 57.1 | 9.1 | 42.1 | 35.9 | 40.0 | 30.0 | 15.4 |
| Financial stress | 34.7 | 21.7 | 27.3 | 41.4 | 33.3 | 57.1 | 18.2 | 21.1 | 43.4 | 33.3 | 30.0 | 53.9 |
| Worried about who will take care of my family if I can't | 33.2 | 0.0 | 31.8 | 44.8 | 35.9 | 42.9 | 27.3 | 42.1 | 35.9 | 26.7 | 40.0 | 53.9 |
| Fear of upcoming procedures | 32.5 | 21.7 | 36.4 | 34.5 | 41.0 | 57.1 | 18.2 | 21.1 | 43.4 | 28.9 | 20.0 | 7.7 |
| Feeling discouraged | 30.6 | 17.4 | 40.9 | 34.5 | 35.9 | 57.1 | 18.2 | 15.8 | 28.3 | 31.1 | 30.0 | 38.5 |
| Feeling disconnected from my family/friends/community support | 28.4 | 34.8 | 36.4 | 37.9 | 20.5 | 28.6 | 27.3 | 26.3 | 30.2 | 22.2 | 10.0 | 38.5 |
| Loneliness | 26.6 | 21.7 | 22.7 | 41.4 | 12.8 | 28.6 | 9.1 | 31.6 | 32.1 | 26.7 | 20.0 | 38.5 |
| Fear of death | 25.8 | 8.7 | 31.8 | 24.1 | 28.2 | 28.6 | 9.1 | 36.8 | 32.1 | 24.4 | 30.0 | 15.4 |
| Difficult to be away from pets | 25.1 | 13.0 | 36.4 | 17.2 | 23.1 | 28.6 | 27.3 | 15.8 | 22.6 | 26.7 | 50.0 | 46.2 |
| Worried about who will take care of me | 24.4 | 21.7 | 36.4 | 24.1 | 30.8 | 14.3 | 27.3 | 26.3 | 24.5 | 17.8 | 10.0 | 23.1 |
| Other family members ill or in trouble | 22.9 | 8.7 | 31.8 | 27.6 | 33.3 | 28.6 | 0.0 | 5.3 | 30.2 | 17.8 | 10.0 | 30.8 |
| **Difficulty accepting how I appear toward others because of my illness*** | 20.7 | 13.0 | 27.3 | 10.3 | 12.8 | 71.4 | 9.1 | 26.3 | 24.5 | 22.2 | 0.0 | 38.5 |
| **Feelings of regret*** | 19.6 | 13.0 | 31.8 | 13.8 | 30.8 | 57.1 | 27.3 | 5.3 | 24.5 | 8.9 | 10.0 | 7.7 |
| No one to talk to about what I'm going through | 15.9 | 8.7 | 13.6 | 24.1 | 12.8 | 0.0 | 18.2 | 21.1 | 20.8 | 8.9 | 10.0 | 30.8 |
| Feeling that others will or are judging me | 15.5 | 17.4 | 22.7 | 20.7 | 15.4 | 14.3 | 9.1 | 15.8 | 17.0 | 13.3 | 0.0 | 7.7 |
| Conflicts with hospital staff | 14.8 | 8.7 | 22.7 | 6.9 | 20.5 | 14.3 | 18.2 | 5.3 | 17.0 | 15.6 | 0.0 | 23.1 |
| Feeling hopeless | 14.0 | 4.4 | 13.6 | 24.1 | 7.7 | 28.6 | 0.0 | 21.1 | 15.1 | 13.3 | 20.0 | 15.4 |
| Feelings of low self-worth | 13.7 | 8.7 | 18.2 | 17.2 | 12.8 | 14.3 | 0.0 | 15.8 | 11.3 | 13.3 | 0.0 | 38.5 |
| Sense of guilt or shame | 13.7 | 13.0 | 18.2 | 20.7 | 5.1 | 14.3 | 0.0 | 5.3 | 15.1 | 13.3 | 10.0 | 38.5 |
| Need for forgiveness | 12.9 | 17.4 | 9.1 | 6.9 | 12.8 | 14.3 | 9.1 | 15.8 | 18.9 | 11.1 | 0.0 | 15.4 |
| Feeling that my suffering is meaningless | 12.2 | 13.0 | 9.1 | 17.2 | 5.1 | 14.3 | 0.0 | 10.5 | 18.9 | 8.9 | 10.0 | 23.1 |
| **Inadequate support from family*** | 11.1 | 4.4 | 9.1 | 13.8 | 10.3 | 42.9 | 18.2 | 15.8 | 17.0 | 0.0 | 0.0 | 15.4 |
| Feelings that I've lost meaning or purpose in life | 10.7 | 8.7 | 18.2 | 17.2 | 7.7 | 14.3 | 9.1 | 5.3 | 13.2 | 8.9 | 0.0 | 7.7 |
| Struggling with disconnection from Higher Power | 10.7 | 8.7 | 13.6 | 10.3 | 10.3 | 14.3 | 9.1 | 10.5 | 9.4 | 2.2 | 20.0 | 38.5 |
| Concerns about the afterlife | 8.9 | 0.0 | 13.6 | 6.9 | 18.0 | 14.3 | 18.2 | 5.3 | 7.6 | 6.7 | 10.0 | 0.0 |
| Questioning my faith | 6.3 | 0.0 | 9.1 | 3.5 | 7.7 | 28.6 | 0.0 | 5.3 | 7.6 | 4.4 | 10.0 | 7.7 |
| Feeling abandoned or punished by God | 5.2 | 4.4 | 4.6 | 3.5 | 0.0 | 14.3 | 9.1 | 15.8 | 5.7 | 0.0 | 10.0 | 15.4 |
| **Anger at God/Higher Power*** | 4.4 | 0.0 | 4.6 | 3.5 | 0.0 | 0.0 | 18.2 | 0.0 | 3.8 | 6.7 | 0.0 | 23.1 |
| Marital troubles | 4.4 | 0.0 | 4.6 | 6.9 | 10.3 | 0.0 | 0.0 | 5.3 | 3.8 | 0.0 | 0.0 | 15.4 |
| STAI Score, mean, SD | 37.3, 16.0 | 32.3, 7.8 | 40.0, 17.4 | 41.1, 20.1 | 35.0, 15.3 | 37.7, 16.5 | 30.9, 15.9 | 32.8, 16.5 | 39.0, 17.6 | 40.3, 14.5 | 35.0, 9.8 | 35.9, 15.3 |
| Total number of stressors endorsed, mean, SD | 10.4, 7.9 | 7.3, 6.1 | 12.7, 6.9 | 11.3, 7.6 | 10.4, 8.0 | 14.7, 11.0 | 7.6, 6.8 | 9.4, 9.2 | 11.6, 9.0 | 9.4, 7.1 | 8.8, 4.6 | 11.6, 9.5 |

Unit Type: Med = general medicine; Neur = Neurology; Card = Cardiology; CV = Cardiovascular; Resp = Pulmonary/Respiratory; Vasc = vascular; Tran = solid organ transplant; Surg = surgical; Onc = hematology/oncology; BMT = bone marrow transplant; Ren = renal/nephrology

* p ≤ .05

** p ≤ .01
