# Supplementary material for: The prevalence, grouping, and distribution of stressors and their association with anxiety among hospitalized patients
Source: PLoS One. 2021 Dec 6;16(12):e0260921. doi: 10.1371/journal.pone.0260921 (PMC8648119; doi:10.1371/journal.pone.0260921)
Supplement: S3 Table — (DOCX) [file pone.0260921.s004.docx]

| **Stressor** | **Mean STAI Score (20-80)** | | **p-value^a^** |
| --- | --- | --- | --- |
|  | **Endorsed** | **Not Endorsed** |  |
| Pain | 40.9 | 32.8 | <.0001 |
| Feeling frustrated | 44.4 | 30.3 | <.0001 |
| Inability to sleep | 42.8 | 32.6 | <.0001 |
| Feeling overwhelmed | 42.2 | 31.9 | <.0001 |
| Fear of the unknown about diagnosis and treatment | 44.5 | 32.0 | <.0001 |
| Loss of physical ability or bodily function | 42.6 | 33.3 | <.0001 |
| Missing out on important events in life | 43.7 | 32.8 | <.0001 |
| Worried about my quality of life | 44.1 | 32.6 | <.0001 |
| Feeling like I've lost control | 46.2 | 32.2 | <.0001 |
| Guilt over being a "burden" to family members | 44.4 | 33.3 | <.0001 |
| Financial stress | 44.1 | 33.7 | <.0001 |
| Worried about who will take care of my family if I can't | 41.9 | 35.0 | .0008 |
| Fear of upcoming procedures | 44.4 | 33.8 | <.0001 |
| Feeling discouraged | 46.9 | 33.0 | <.0001 |
| Feeling disconnected from my family, friends, communities of support | 45.1 | 34.2 | <.0001 |
| Loneliness | 48.3 | 33.3 | <.0001 |
| Fear of death | 45.3 | 34.5 | <.0001 |
| Difficult to be away from pets | 44.2 | 35.0 | .0002 |
| Worried about who will take care of me | 46.5 | 34.3 | <.0001 |
| Other family members ill or in trouble | 44.3 | 35.2 | .0003 |
| Difficulty accepting how I appear toward others because of my illness | 46.6 | 34.9 | <.0001 |
| Feelings of regret | 46.2 | 35.1 | <.0001 |
| No one to talk to about what I'm going through | 48.8 | 35.1 | <.0001 |
| Feeling that others will or are judging me | 48.4 | 35.2 | <.0001 |
| Conflicts with hospital staff | 44.3 | 36.1 | .0074 |
| Feeling hopeless | 52.4 | 34.8 | <.0001 |
| Feelings of low self-worth | 51.6 | 35.0 | <.0001 |
| Sense of guilt or shame | 54.7 | 34.5 | <.0001 |
| Need for forgiveness | 48.4 | 35.6 | <.0001 |
| Feeling that my suffering is meaningless | 50.0 | 35.5 | <.0001 |
| Inadequate support from family | 50.1 | 35.7 | <.0001 |
| Feelings that I've lost meaning or purpose in life | 52.7 | 35.4 | <.0001 |
| Struggling with disconnection from Higher Power | 49.4 | 35.8 | <.0001 |
| Concerns about the afterlife | 45.6 | 36.5 | .0017 |
| Questioning my faith | 51.0 | 36.4 | .0003 |
| Feeling abandoned or punished by God | 46.7 | 36.8 | .0425 |
| Anger at God/Higher Power | 53.9 | 36.5 | .0007 |
| Marital troubles | 51.1 | 36.6 | .0054 |

STAI = State-Trait Anxiety Index, 6-item scale prorated to 20-80 range

^a^T-test for stressors endorsed n ≥ 30, Mann-Whitney U test for stressors not normally distributed and endorsed n < 30; all are significant at .05 level
